# Assembly of the Complete Mitochondrial Genome of *Gelsemium elegans* Revealed the Existence of Homologous Conformations Generated by a Repeat Mediated Recombination

**DOI:** 10.3390/ijms24010527

**Published:** 2022-12-28

**Authors:** Chuihuai You, Tianzhen Cui, Chang Zhang, Shoujian Zang, Yachun Su, Youxiong Que

**Affiliations:** 1 College of Life Sciences, Fujian Agriculture and Forestry University, Fuzhou 350002, China; 2 Key Laboratory of Sugarcane Biology and Genetic Breeding, Ministry of Agriculture and Rural Affairs, Fujian Agriculture and Forestry University, Fuzhou 350002, China

**Keywords:** *Gelsemium elegans*, mitochondrial genome, homologous recombination, phylogenetic analysis, RNA editing events

## Abstract

*Gelsemium elegans* (*G. elegans*) is a Chinese medicinal plant with substantial economic and feeding values. There is a lack of detailed studies on the mitochondrial genome of *G. elegans*. In this study, the mitochondrial genome of *G. elegans* was sequenced and assembled, and its substructure was investigated. The mitochondrial genome of *G. elegans* is represented by two circular chromosomes of 406,009 bp in length with 33 annotated protein-coding genes, 15 tRNA genes, and three rRNA genes. We detected 145 pairs of repeats and found that four pairs of repeats could mediate the homologous recombination into one major conformation and five minor conformations, and the presence of conformations was verified by PCR amplification and Sanger sequencing. A total of 124 SSRs were identified in the *G. elegans* mitochondrial genome. The homologous segments between the chloroplast and mitochondrial genomes accounted for 5.85% of the mitochondrial genome. We also predicted 477 RNA potential editing sites and found that the *nad4* gene was edited 38 times, which was the most frequent occurrence. Taken together, the mitochondrial genome of *G. elegans* was assembled and annotated. We gained a more comprehensive understanding on the genome of this medicinal plant, which is vital for its effective utilization and genetic improvement, especially for cytoplasmic male sterility breeding and evolution analysis in *G. elegans*.

## 1. Introduction

*Gelsemium elegans* (Gardner and Champ.) Benth. (China National Center for Biotechnology Information Taxonomy ID: 427660) [1], also known as Da Cha Yao, Duan Chang Cao, or Pig Ginseng, is a perennial evergreen vine of the genus *Gelsemium* in Loganiaceae. It is a medicinal plant with substantial economic and feeding value. The whole *G. elegans* plant can be used as medicine [2]. In the farming industry, *G. elegans* acts in boosting the immunity, deworming, and promoting appetite, improving livestock survival and growth rates [3]. Broiler chickens and pigs fed with a certain amount of *G. elegans* additive feed have increased resistance, significant growth, and weight gain, as well as reduced meat ratio [4]. As a Chinese herbal medicine, *G. elegans* has immunomodulatory, neuropathic analgesic, anti-inflammatory, antianxiety, and antitumor effects [5,6,7]. Up to now, more than 200 compounds have been isolated and identified from *G. elegans*, mainly including steroids, iridoids, and indole alkaloids [8,9]. Indole alkaloids are significant active components for the function of *G. elegans* and have received much attention in drug development [10]. With the development of molecular biology, there has also been some progress related to the genome of *G. elegans* [1], which lays the foundation for elucidating the biosynthesis of *G. elegans* secondary metabolites and functional gene mining.

Mitochondria are essential organelles in eukaryotic cells. The endosymbiosis theory postulates the production of mitochondria from an endosymbiotic alphaproteobacterium within an archaeal-derived host cell, which eventually evolved into organelles of eukaryotic cells [11]. They can be essential for studying the origin, phylogeny, and genetic diversity of a certain species [12]. As semi-autonomous organelles, mitochondria contain a separate mitochondrial genome [13]. The mutation rate of mitochondrial genome in plants is much lower than that in their nuclei or chloroplasts [14]. As reported, plant mitochondrial genomes possess many unique features [15] and exhibit a complex and dynamic structure with a large amount of unconserved DNA of unknown function [16]. The structure is usually circular, but it is more complex in vivo [17]. In some species, the mitochondrial genomes have linear or even multichromosomal structures [15,18]. Moreover, they contain abundant repetitive sequences, which can lead to rapid genome rearrangement [19]. The above characteristics of the mitochondrial genome provide helpful information for evolutionary and phylogenetic studies [20]. Higher plant mitochondria provide energy material for plant growth and participate in the synthesis and metabolism of amino acids, lipids, vitamins, and other substances related to life activities, playing an essential role in plant growth and development [21,22,23]. A total of 9243 chloroplast genomes and 1240 plastid genomes are included in the NCBI database, but the number of plant mitochondrial genomes is only 545 (https://www.ncbi.nlm.nih.gov/genome/browse#!/overview/) (accessed on 14 November 2022). The *G. elegans* mitochondrial genome was reported in 2019 [24]. However, due to the limitation of sequencing technology and one-sided analysis by the authors at that time, the study of the G. elegans mitochondrial genome is still half-understood. There are still some problems to be improved. Firstly, the assembly quality of the published *G. elegans* mitochondrial genome is low, and the repetitive regions of this assembly are randomly selected; thus, the probability of genomic structural errors is high. Secondly, there is no annotation information. Thirdly, there is a lack of systematic research. The published mitochondrial genomes only report the length, GC content, and the number of genes in *G. elegans* mitochondria, but lack comprehensive information on mitochondrial genome codon preference, repetitive sequence, phylogeny, sequence migration, and RNA editing events. At present, the plant trait of cytoplasmic male sterility (CMS) in *G. elegans* has not yet been reported. CMS is determined by the mitochondrial genome and is associated with the pollen sterile phenotype [25]. Mitochondrial genomes also contain open reading frames (ORFs) of unknown function, some of which are essential genes responsible for CMS in plants [26,27,28]. In addition, RNA editing is a post-transcriptional horizontal modification that changes the mRNA nucleotides generated by gene transcription, leading to the phenomenon that its sequence is not entirely consistent with the template sequence, an essential feature of animal and plant mitochondria and plant chloroplast genomes [29]. Many intramolecular recombination events and subgenomic conformations have been reported in plant mitochondria [30]. Therefore, as an important medicinal plant, an in-depth study of the mitochondrial genome of *G. elegans* is of great significance for its effective utilization and genetic improvement.

In this study, we sequenced, assembled, and annotated the mitochondrial genome of *G. elegans* and investigated its genomic and structural features. The presence of its mitochondrial genomic substructure was explored through PCR experiments and nanopore reads. To better reveal the homologous fragments of the *G. elegans* mitochondrial and chloroplast genomes, we assembled its chloroplast genes using the same data set and completed the sequence similarity analysis. Moreover, the mitochondrial genome for RNA editing events was analyzed. This study further confirms the existence of multiple conformations in plant mitochondrial genomes, providing a theoretical basis for cytoplasmic male sterility breeding and plant evolution in higher plants.

## 2. Results

### 2.1. General Features of G. elegans Mitochondrial Genome

**Figure 1:**
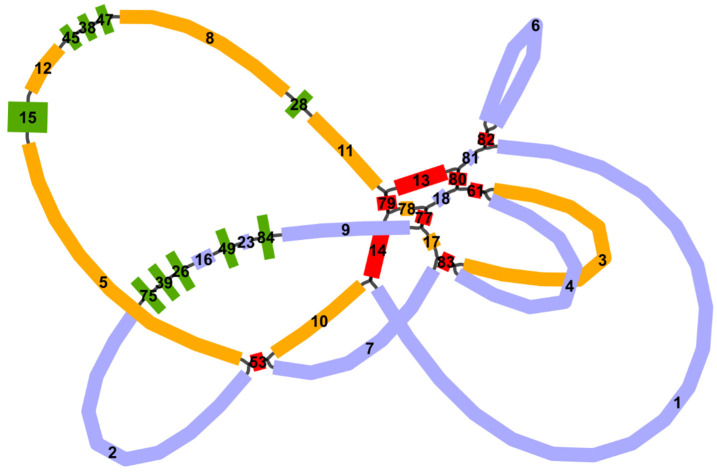
The mitochondrial genome of *G. elegans* according to a graph model. The purple contigs belong to chromosome 1. The yellow contigs belong to chromosome 2. The green contigs are the mitochondrial plastid sequences (MTPTs), in which contigs 26, 39, 49, 75, and 84 belong to chromosome 1, and contigs 15, 28, 38, 45, and 77 belong to chromosome 2. The red contigs are the repeat regions, in which contig 82 belongs to chromosome 1, and contigs 13, 14, 53, 61, 77, 79, 80, and 83 belong to both chromosomes.

**Figure 2:**
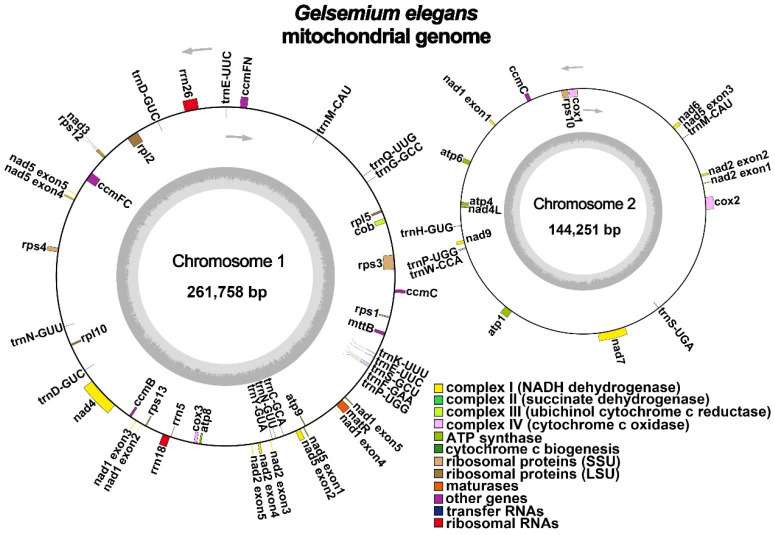
The circular maps of the mitochondrial genomes of *G. elegans*. Genomic features transcribed clockwise and counterclockwise are drawn on the inside and outside of the circle, respectively. Color-coding is used to distinguish genes of different functional groups.

The *G. elegans* mitochondrial genome was sequenced, assembled, and annotated. After resolving the repeat regions, the graph-based mitochondrial genome containing 37 nodes was established, as shown in Figure 1. We found that the *G. elegans* mitochondrial genome (NCBI Accession numbers: OP805353 and OP805354) has multiple “chromosomes” (Figure 2) with a total length of 406,009 bp and a GC content of 44.43%. The length of chromosomes 1 and 2 was 261,758 bp and 144,251 bp, respectively. The specific solution paths are shown in Table S2.

There were 51 genes annotated in the mitochondrial genome of *G. elegans*, including 33 protein-coding genes (PCGs), 15 tRNA genes (five tRNAs were multiple copies), and three rRNA genes (Table 1). These 33 PCGs could be divided into nine classes (Table 1), including ATP synthase (five genes), NADH dehydrogenase (nine genes), ubiquinol cytochrome c reductase (four genes), cytochrome C oxidase (three genes), transport membrane protein (one gene), maturation enzyme (one gene), cytochrome c biogenesis (one gene), ribosomal large subunit (three genes), and ribosomal small subunit (six genes).

### 2.2. Repeat Sequence Analysis

**Figure 3:**
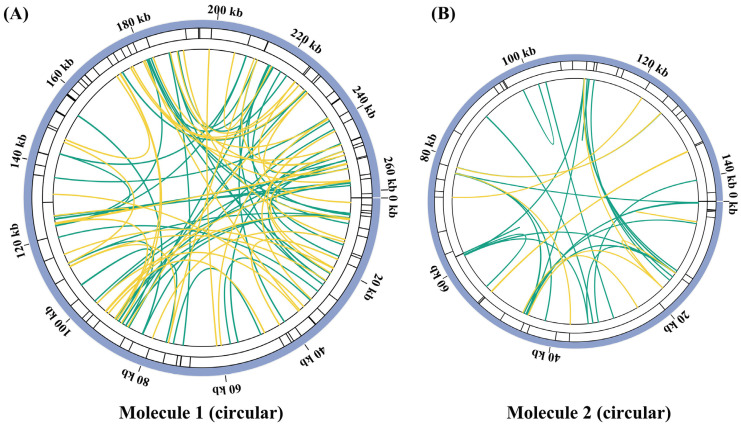
Repeat analysis of the mitochondrial genome in *G. elegans*. (**A**,**B**) Repeated sequences were analyzed for each of the two chromosomes. The colored lines on the innermost circle connect the two repetitive sequences of the scattered repeats, with the yellow line representing palindromic match and the green line representing forward match. The black line on the second circle represents the tandem repeat sequence, and the black line on the outermost circle represents the microsatellite or simple sequence repeat (SSR) sequence.

Microsatellites are also known as simple repeat sequences (SSRs). Here, 82 SSRs were identified in chromosome 1 of the *G. elegans* mitochondrial genome, and their proportion of different forms is shown in Figure 3 and Table S3. Monomeric and dimeric forms of SSRs accounted for 51.22% of the total SSRs (Table S3). Adenine (A) monomer repeat accounted for 51.85% (14) of 27 monomer SSRs, and TA repeat was the most common type among the dimeric SSRs, accounting for 33.33%. Two pentameric and one hexameric SSRs were found in chromosome 1. Furthermore, 42 SSRs were observed in chromosome 2 of the *G. elegans* mitochondrial genome (Figure 3, Table S3), with monomeric and dimeric forms accounting for 50.00% (21) of the total SSRs. Adenine (A) monomer repeat accounted for 69.23% (9) of the 13 monomer SSRs, and TA repeat was also the most frequent type among the dimeric SSRs, accounting for 37.50%. There were no pentameric and hexameric SSRs in chromosome 2 (Table S3). These above SRRs could be potential identification markers for fingerprinting *G. elegans*.

Tandem repeats are also known as satellite DNA. As shown in Table S4, a total of six tandem repeats with a length ranging from 18 to 25 bp and the proportion of similarity of each unit from 78% to 86% (except one 66%) were present in chromosome 1 of *G. elegans* mitochondrial genome. In chromosome 2, a total of five tandem repeats with a length ranging from 15 to 27 bp and the proportion of similarity of each unit from 84% to 96% were observed (Figure 3 and Table S4).

The dispersed repeats in chromosomes 1 and 2 were examined. In chromosome 1, 104 repetitive sequences with lengths greater than or equal to 30 bp were observed, including 60 pairs of forward repetitive sequences and 44 pairs of palindromic repetitive sequences, with the longest direct repeat of 102 bp. Chromosome 2 contained 28 pairs of forward repetitive sequences and 13 pairs of palindromic repetitive sequences, with the longest direct repeat of 163 bp (Figure 3, Table S5).

### 2.3. Repeat-Mediated Homologous Recombination

Previous studies have demonstrated that repeat sequences could mediate homologous recombination in mitochondrial genomes [31]. There were four repeat sequences that could potentially mediate the homologous recombination in *G. elegans* mitochondrial genome supported by long reads. They were named R1, R2, R3, and R4 (Table 2). Their lengths were 9816 bp, 360 bp, 211 bp, and 137 bp, respectively. Among them, R1, R3, and R4 were forward repeats, while R2 was a reverse repeat. 

**Figure 4:**
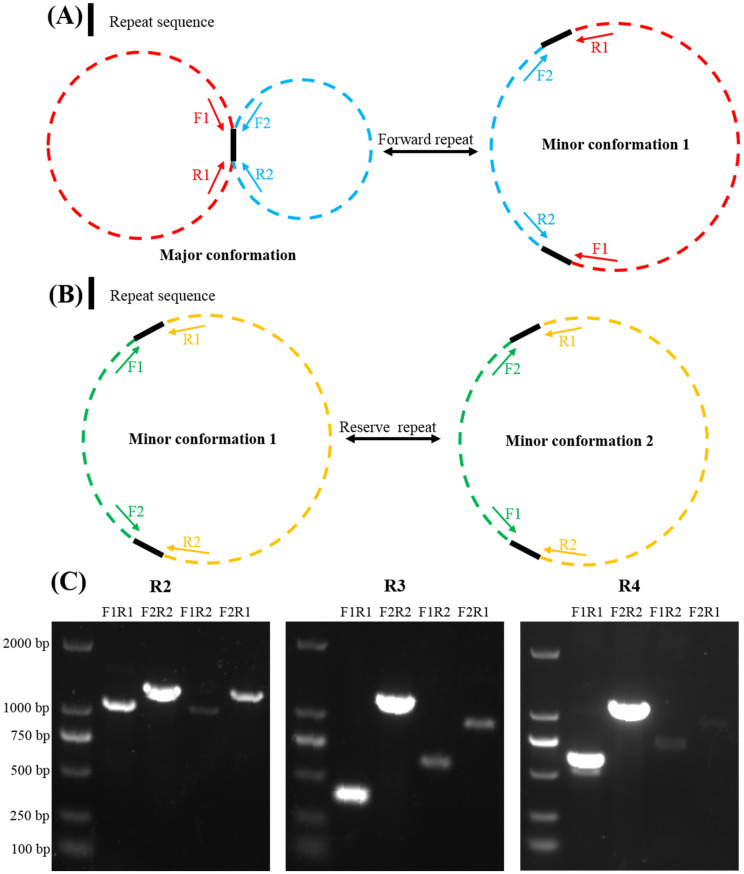
Validation of the homologous recombination mediated by different pair repeats. (**A**,**B**) The schematic diagram of primer design and experimental design after reverse primer exchange of direct repeat sequences that can mediate homologous recombination in representative circular molecules. (**C**) PCR validation results of the existence of various conformations of mitochondrial DNA. From left to right, each of the five lanes represents a set of three experiments, namely, repeat2, repeat3, and repeat4. The experiments are as follows, from left to right: marker, major conformation 1, major conformation 2, minor conformation 1, and minor conformation 2.

To further verify whether these four repeats can mediate homologous recombination, PCR amplification and Sanger sequencing were performed to validate the existence of the minor conformation. The scheme of the primer design was shown in Figure 4. We selected three short repeat sequences, R2, R3, and R4, for primer design and validation. Both pairs of specific primers, i.e., primer F1/primer R1 and primer F2/primer R2, could amplify repeat sequences. We swapped the reverse primers, i.e., primer F1/primer R2 and primer F2/primer R1, and found that they could amplify repeat sequences (Figure 4). All PCR products were validated by Sanger sequencing to prove the authenticity of both conformations, which is consistent with the results we obtained on the basis of the long-read analysis (Figure 4).

**Figure 5:**
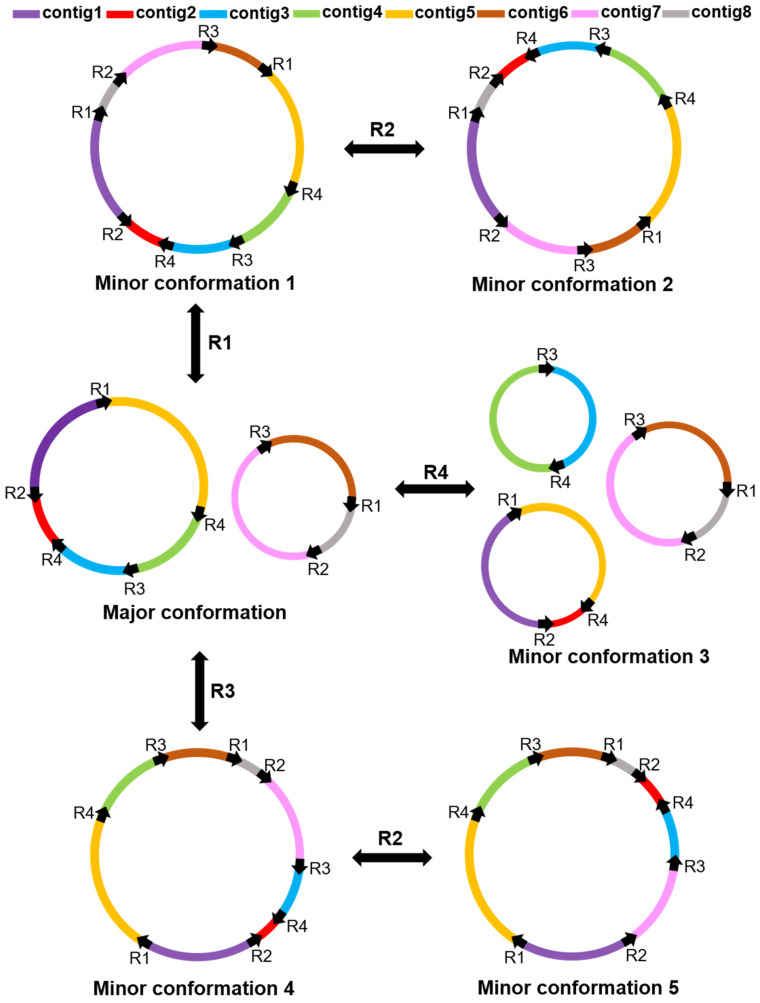
Hypothetical products generated by recombinations mediated by R1, R2, R3, and R4. R1, R2, R3, and R4 in the picture represent repeats 1–4. The black arrows on the circular chromosome represent the repeat sequences, and the colored lines represent the DNA fragment between the repeats. The circles represent the mitochondrial genome conformations. Minor conformations 1, 2, 4, and 5 contain one circular chromosome, and the major conformation contains two circular chromosomes, while minor conformation 3 contains three circular chromosomes.

According to the validation results, we could speculate the potential homologous recombination type of the *G. elegans* mitochondrial genome (Figure 5). The major conformations of the *G. elegans* mitochondrial genome were two circular chromosomes, which could form a separate circular chromosome by R1 or R3 (minor conformations 1 and 2). On this basis, contig 1 and contig 8 of the independent circular chromosome minor conformation 1 and contig 7, contig 3, and contig 2 of the independent circular chromosome minor conformation 4 could form minor conformations 2 and 5 through repetitive sequence R2-mediated inversion. In addition to this, contig 4 and contig 3 of chromosomes could form the independent circular chromosome by repeating sequence R4. The entire mitochondrial genome of *G. elegans* formed three independent circular chromosomes. To sum up, the four pairs of repeats had mediated genome recombination, forming one major conformation and five minor conformations.

### 2.4. Identification of MTPTs

**Figure 6:**
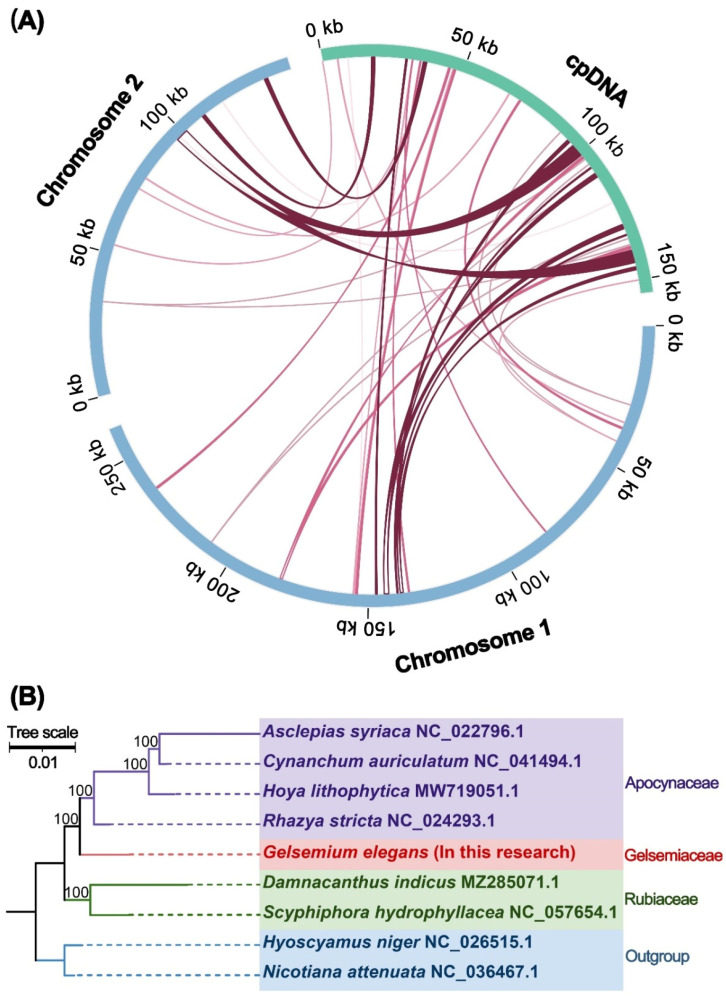
Homologous fragments and phylogenetic analysis of *G. elegans*. (**A**) The blue arcs represent the mitochondrial genome, the green arcs represent the chloroplast genome, and the red lines between the arcs correspond to homologous genomic fragments. (**B**) The evolutionary relationship between *G. elegans* and homologous species.

In the previous annotation of the organelle genome, it was found that there was extensive sequence migration between the plastid and mitochondria, and we called these mitochondrial plastid sequences (MTPTs). The mitochondrial genome sequence of *G. elegans* was compared with its plastid genome, and 37 matching points between the two organelle genomes were identified (Figure 6A, Table S6). A total of 37 fragments were homologous to the mitochondrial and chloroplast genomes between chromosome 1 and chromosome 2, with a total length of 23,747 bp, accounting for 5.85% of the total mitochondrial genome length. Four of the fragments exceeded 1000 bp, with fragments 1 and 2 being the longest at 1578 bp. By annotating these homologous sequences, eight complete genes were also identified on 37 homologous fragments, including two PCGs (*petN* and *rps7*) and six tRNA genes (*trnN-GUU*, *trnD-GUC*, *trnS-GGA*, *trnS-GCU*, *trnW-CCA*, and *trnH-GUG*). These six tRNA sequences from the chloroplast and mitochondrial genomes remain similar, indicating that they may still function in the mitogenome.

### 2.5. Phylogenetic Analysis

To further explore the evolutionary relationships of *G. elegans* mitochondria, we constructed a phylogenetic tree (Figure 6B) using the DNA sequences of 22 conserved mitochondrial PCGs (*atp1*, *atp6*, *atp8*, *ccmC*, *ccmFc*, *cob*, *cox2*, *cox3*, *nad1*, *nad2*, *nad5*, *nad6*, *nad7*, *nad9*, *matR*, *rpl16*, *rps1*, *rps3*, *rps4*, *rps13*, *rps19*, and *sdh4*) from nine species (*G. elegans*, OP805353 and OP805354; *Damnacanthus indicus*, MZ285071; *Scyphiphora hydrophyllacea*, NC057654; *Hoya lithophytica*, MW719051; *Cynanchum auriculatum*, NC041494; *Asclepias syriaca*, NC022796; *Rhazya stricta*, NC024293; *Nicotiana attenuate*, NC036467; *Hyoscyamus niger*, NC026515). Two mitochondrial genomes of *Solanaceae* species were set as outgroups. The topology of the mitochondrial DNA-based phylogeny coincided with the latest classification of the Angiosperm Phylogeny Group (APG). *G. elegans* belongs to the family *Gelsemiaceae* and is more closely related to *R. stricta*.

### 2.6. RNA Editing Events in G. elegans

**Figure 7:**
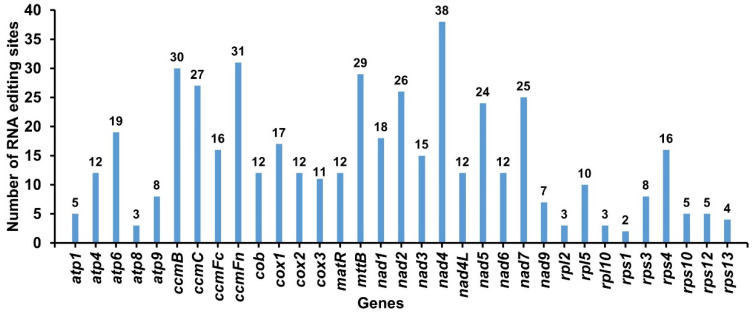
Prediction of RNA editing events in *G. elegans* mitochondrial genome. The horizontal and vertical coordinates indicate the gene name and the number of RNA edits, respectively.

RNA editing events were identified for 33 PCGs from *G. elegans* mitochondria according to predictions from the online website PREP suit (http://prep.unl.edu/) (accessed on 6 July 2022). The setting standard was a cutoff value = 0.2. Under this criterion, a total of 477 potential RNA editing sites were identified on 33 mitochondrial PCGs (Figure 7), all of which were C-to-U base editing. The *nad4* gene possessed 38 potential RNA editing sites of the mitochondrial genes, which was the most edited of all mitochondrial genes. This was followed by the *ccmFn* gene with 31 RNA editing events. The *rps*1 gene had only two potential RNA editing events, and the number of edits was the lowest among all mitochondrial genes.

## 3. Discussion

### 3.1. Multiple Circular Conformations in Plant Mitochondrial Genome

Mitochondria are the powerhouse of plants and provide the energy required for plant growth and development. Plant mitochondrial genomes are more complex than animal mitochondria, and they exhibit extensive variation in size (191–11,319 kb), sequence arrangement, and repeat content [16]. In this study, the mitochondrial genomic characteristics of *G. elegans*, a traditional Chinese medicine with important medicinal and feeding values, were investigated. According to previous studies, most mitochondrial genomic structures are circular; for example, the kiwifruit mitochondrial genome is two widely differentiated circular chromosomes [32]. In contrast, the lettuce mitochondrial genome structure is composed of multiple linear, branched, and circular structures [16]. Wu et al. [15] found that about 10% of the sequenced plant mitogenome has multiple chromosome structures. In eukaryotes, mitochondria are diverse in genome structure, including the repeated evolution of multichromosomes [18]. In the present study, the *G. elegans* mitochondrial genome was represented by two circular chromosomes (Figure 1) with a size of 406,009 bp, which is in accordance with the two circular mitogenome chromosomes of *Actinidia chinensis* [32] and *Scutellaria tsinyunensis* [33]. However, the cucumber mitochondrial genome has three separate chromosomes [34]. The mitogenome of *Rhopalocnemis phalloides* consists of 21 minicircular chromosomes [35]. Therefore, in-depth studies of plant multichromosomal mitochondrial genomes will contribute to new discoveries in their genome evolution and molecular function.

### 3.2. Repeated Sequence-Mediated Homologous Recombination

Repeated sequences are widely present in the mitochondrial genome, mainly including tandem, short, and large repeats [36]. Previous studies have shown that repetitive sequences in mitochondria are essential for intermolecular recombination [37]. In the *Prunus salicina* mitogenome, three pairs of repetitive sequences were found to mediate the genome recombination into eight different conformations [38]. Similarly, three repetitive sequences in *I. batatas* were shown to mediate the presence of seven conformations [31]. In addition, a pair of repeat sequences in the *S. tsinyunensis* mitogenome could mediate two conformations [33]. SSRs are often used for molecular marker design due to their high polymorphism [39], which is a repeat of a DNA fragment consisting of short sequence units with a length of 1–6 base pairs [40]. Tandem repeats represent core repeating units of about 1–200 bases repeated multiple times in a tandem fashion [41]. They are widely found in eukaryotic genomes and some prokaryotes. The SSRs, tandem repeats, and dispersed repeats were also investigated in our study (Figure 3, Tables S3–S5). The *G. elegans* mitochondrial genome contains abundant repetitive sequences, which may indicate frequent intermolecular recombination in the mitochondrial genome, with dynamically altered sequence and conformation during evolution. The four pairs of repetitive sequences identified in this study could mediate the formation of one major and five minor conformations in the *G. elegans* mitochondrial genome (Figure 5). Among them, the minor conformation 3 mediated by R4 had three independent circular molecules (Figure 5). Specific DNA repair in the plant mitochondrial genome may be related to these phenomena [42]. We only verified the existence of these conformations, but what functions they perform still needs further investigation. The results of this study further support the existence of multiple conformations in the mitotic genome of plants. 

### 3.3. Phylogenetic Relationships of Homologous Species

The rapid evolution of plant mitochondria has led to large-scale gene recombination, genomic heterogeneity, and gene chimerism in the mitochondria of different species [43]. Changes in the size and structure of plant mitochondrial genomes are evident, but functional genes remain conserved [44]. Here, we constructed a sequence-based phylogenetic tree using PCGs to explore the evolutionary relationships between *Gelsemium* and the representative taxa of angiosperms (Figure 6B). It clearly reflects the transparent taxonomic relationships among these taxa, and the results also show that *G. elegans* is more closely related to *R. stricta*. 

### 3.4. Prediction of RNA Editing Sites

RNA editing is a post-transcriptional process. In higher plants, mitochondrial RNA editing is associated with potential physiological processes and molecular functions [45,46]. In particular, the imprecise repair mechanisms in mitochondrial genomes often lead to the insertion of foreign DNA sequences into the genome, especially plastid DNA [33]. Prediction of potential RNA editing sites helps us understand the expression of plant mitochondrial genes. Previous studies reported 491 potential RNA editing sites in 34 genes of rice [47] and 216 potential RNA editing sites in 26 genes of *S. glauca* [41]. In the present study, 477 potential editing sites were predicted in 33 genes, all of which were C-to-U editing. The prediction and characterization of potential RNA editing sites provide essential clues for predicting gene function using new codons.

### 3.5. Intracellular Sequence Transfer Events in G. elegans Mitogenome

As the number of sequenced genomes of mitochondrial and chloroplast increases, studies on the diversity and hotspots of MTPT transfer becomes feasible. The discovery of MTPTs can contribute to the accurate assembly of chloroplast and mitochondrial genomes and to the understanding of organelle genome evolution [48]. During plant evolution, some chloroplast fragments can migrate into the mitochondrial genome. We found that 37 fragments, which were likely MTPT sequences, migrated from the chloroplast genome to the mitochondrial genome (Figure 6A, Table S6). These sequences were 23,747 bp in length, accounting for 5.85% of the mitogenome. Similarly, 32 MTPTs with a total length of 26,870 kb were found in *S. glauca* [41], accounting for 5.18% of the mitogenome. The MTPTs, which were also found in *S. tsinyunensis*, represented 0.95% (3372 bp) of the mitogenome [33]. Previous studies have found that, in angiosperms, tRNA genes occasionally migrated from the chloroplast to mitochondria [20]. The six homologous fragments from the chloroplast of *G. elegans* were complete tRNA genes (Table S6), suggesting that they may play a role in normal functions [49]. 

## 4. Materials and Methods

### 4.1. Plant Materials, DNA Extraction, and Sequencing

Five fresh leaves in the same *G. elegans* plant were collected from Yongding District, Fujian Province (GPS: 116°54′ E, 24°24′ N; altitude 602 m). The samples were stored in the Fujian Key Laboratory of Sugarcane Biology and Genetic Breeding, Ministry of Agriculture and Rural Affairs, Fujian Agriculture and Forestry University. High-quality genomic DNA was extracted using the CTAB method [50].

The DNA library with an insert size of 300 bp was constructed using a Nextera DNA Flex Library Prep Kit (Illumina, San Diego, CA, USA) and sequenced using the Illumina NovaSeq platform (Illumina, San Diego, CA, USA). The libraries were prepared for Oxford Nanopore sequencing using the SQK-LSK109 ligation kit and the standard protocol. The purified library was loaded onto primed R9.4 Spot-On Flow Cells and sequenced using a PromethION sequencer (Oxford Nanopore Technologies, Oxford, UK) with 48 h runs at Wuhan Benagen Tech Solutions Company Limited (Wuhan, China). Base calling analysis of raw data was performed using the Oxford Nanopore GUPPY software (v0.3.0).

### 4.2. Genome Assembly, Modification and Annotation

The *G. elegans* mitochondrial genome was assembled using a hybrid Illumina and Nanopore strategy. The mitochondrial genome assembly of *G. elegans* was performed using the default parameters (v1.7.5) of GetOrganelle software [51] on Illumina data to obtain a graph-based plant mitochondrial genome. The graph-based plant mitochondrial genome was visualized using Bandage software [52], and the single contig of the chloroplast and nuclear genomes were manually removed. The Nanopore data were then compared to the graphical mitochondrial genome fragments using bwa software [53]. The obtained Nanopore data were used to resolve repetitive sequence regions of the graph-based plant mitochondrial genome of *G. elegans*. The *G. elegans* mitochondrial genome’s circular chromosome was obtained after the contigs were merged by Bandage software [52]. The chloroplast genome was assembled by the GetOrganelle software with the default parameters using Illumina data [51]. 

The mitochondrial genome was annotated using Geseq software [54]. The tRNA and rRNA of mitochondrial genomes were annotated using tRNAscan-SE [55] and BLASTN software [56]. The chloroplast genome was annotated using CPGAVAS2 with database 2 [57]. The annotation errors of each mitochondrial genome were manually corrected using Apollo software [58]. The identified transferred DNA segments were also annotated using GeSeq.

### 4.3. DNA Repeat Sequence Analysis

MISA (https://webblast.ipk-gatersleben.de/misa/) (accessed on 6 July 2022) [59], TRF (https://tandem.bu.edu/trf/trf.unix.help.html) (accessed on 6 July 2022) [60], and the REPuter web server (https://bibiserv.cebitec.uni-bielefeld.de/reputer/) (accessed on 6 July 2022) [61] were used to identify repetitive sequences, including microsatellite sequence repeats, tandem repeats, and dispersed repeats. The results were visualized using the Circos package [62]. Protein-coding sequences of the genome were extracted by Phylosuite software [63]. 

### 4.4. Repeat-Mediated Homologous Recombination Predication and PCR Amplification Validation

BLASTN was used to detect paired repeats and to identify potential recombinations from the repeats. Each pair of repeats and their adjacent 1000 bp sequences were extracted as two templates. We then swapped the adjacent 1000 bp sequences and recombined them to form the other two conformations. The long reads were mapped to the template sequences to check which one could be repeated across a total of four template sequences. We selected three of them for PCR validation. The primers at the ends of repeats were designed by Primer-BLAST (https://www.ncbi.nlm.nih.gov/tools/primer-blast/index.cgi?LINK_LOC=BlastHome) (accessed on 6 July 2022) (Table S1). The PCR amplification system was 50 μL in total, consisting of 2 μL of template gDNA, 2 μL of forward primer, 2 μL of reserve primer, 25 μL of 2× Rapid Taq Master Mix, and 19 μL of ddH_2_O. The PCR amplification program was 95 °C denaturation for 3 min, 35 cycles of 95 °C for 15 s, 55 °C for 15 s, and 72 °C for 30 s, and a 72 °C final extension for 15 min.

### 4.5. Phylogenetic Analysis and RNA Editing Site Prediction

The mitochondrial genomes of eight species were selected for phylogenetic analysis, with *N. attenuate* (Mitochondrion: NC_036467.1) and *H. niger* (Mitochondrion: NC_026515.1) being set as outlier groups. The mitochondrial genomes of these nine species were downloaded from the NCBI database, the shared genes were extracted using PhyloSuite software [63]. The multiple sequence alignment analysis was performed using MAFFT software [64], followed by phylogenetic analysis using MRBAYES software [65]. The results of the phylogenetic analysis were visualized using ITOL software [66]. The prediction of RNA editing events was performed through the online website PREP suit (http://prep.unl.edu/) (accessed on 6 July 2022) [67].

## 5. Conclusions

We successfully assembled the mitochondrial genome of *G. elegans* using a mixed strategy of Illumina and Nanopore. The *G. elegans* mitochondrial genome was two circles with 406,009 bp. A total of 51 genes were annotated in the mitochondrial genome of *G. elegans*, including 33 PCGs, 15 tRNA genes, and three rRNA genes. The *G. elegans* mitochondrial genome contained three forward repeats (9816 bp, 211 bp, and 137 bp) and one pair of reverse repeats (360 bp) that mediate homologous recombination, resulting in the formation of one major conformation and five minor conformations. This study provides valuable information for a better understanding of the genetics of *Gelsemium* and other higher plants.

## Figures and Tables

**Table 1:** Gene composition in the mitogenome of *G. elegans*.

Group of Genes	Name of Genes
ATP synthase	*atp*1, *atp*4, *atp*6, *atp*8, *atp*9
NADH dehydrogenase	*nad*1, *nad*2, *nad*3, *nad*4, *nad*4L, *nad*5, *nad*6, *nad*7, *nad*9
Cytochrome c biogenesis	*cob*
Ubiquinol cytochrome c reductase	*ccm*B, *ccm*C (×2), *ccm*FC, *ccm*FN
Cytochrome c oxidase	*cox*1, *cox*2, *cox*3
Maturases	*mat*R
Transport membrane protein	*mtt*B
Large subunit of ribosome	*rpl*2, *rpl*5, *rpl*10
Small subunit of ribosome	*rps*1, *rps*3, *rps*4, *rps*10, *rps*12 (×2), *rps*13
Ribosome RNA	*rrn*5, *rrn*18, *rrn*26
Transfer RNA	*trn*C-GCA, *trn*D-GUC (×2), *trn*E-UUC (×2), *trn*F-GAA, *trn*G-GCC, *trn*H-GUG, *trn*K-UUU, *trn*M-CAU (×2), *trn*N-GUU (×2), *trn*P-UGG (×2), *trn*Q-UUG, *trn*S-GCU, *trn*S-UGA, *trn*W-CCA, *trn*Y-GUA

**Table 2:** List of four repeat sequences mediated homologous recombination in the mitogenome of *G. elegans*.

Repeat Name	Repeat 1	Repeat 2	Repeat 3	Repeat 4
Identities (%)	100	100	98.65	91.24
Length (bp)	9816	360	211	137
Position-1	251,943–261,758	190,267–190,626	125,011–125,232	82,546–82,676
Position-2	38,228–48,043	60,101–59,742	142,600–142,820	155,253–155,389
E-value	0	0	6.90 × 10^−108^	1.63 × 10^−44^
Type	Forward	Reserve	Forward	Forward

## Data Availability

The mitogenome sequences were deposited on NCBI (https://www.ncbi.nlm.nih.gov) (accessed on 11 November 2022) with accession numbers of OP805353 and OP805354. The sample was deposited in the Fujian Key Laboratory of Sugarcane Biology and Genetic Breeding, Ministry of Agriculture and Rural Affairs, Fujian Agriculture and Forestry University, Fuzhou, China, with voucher number: 20210506.

## Supplementary Materials

The following supporting information can be downloaded at: https://www.mdpi.com/article/10.3390/ijms24010527/s1.

## References

[1] Liu Y.S., Tang Q., Cheng P., Zhu M.F., Zhang H., Liu J.Z., Zuo M.T., Huang C.Y., Wu C.Q., Sun Z.L. (2020). Whole-genome sequencing and analysis of the Chinese herbal plant *Gelsemium elegans*. Acta Pharm. Sin. B.

[2] Lin H.L., Qiu H.Q., Cheng Y., Liu M., Chen M.H., Que Y.X., Que W.C. (2021). *Gelsemium elegans* Benth: Chemical components, pharmacological effects, and toxicity mechanisms. Molecules.

[3] Wang Y., Li Y.W., Sun Z.L., Li Y.Y., Chen X.J. (2016). Research progress on chemical constituents and pharmacological effects of *Gelsemium elegans* Benth. J. Tradit. Chin. Med..

[4] Wu J.W. (2015). Study on the Promotion of Growth in Pigs by *Gelsemiumelegans* (Gardncechamp) Benth. Ph.D. Thesis.

[5] Cai J., Lei L.S., Chi D.B. (2009). Antineoplastic effect of koumine in mice bearing H22 solid tumor. J. South. Med. Univ..

[6] Xu Y., Qiu H.Q., Liu H., Liu M., Huang Z.Y., Yang J., Su Y.P., Yu C.X. (2012). Effects of koumine, an alkaloid of *Gelsemium elegans* Benth., on inflammatory and neuropathic pain models and possible mechanism with allopregnanolone. Pharmacol. Biochem. Behav..

[7] Que W.C., Wu Z.Y., Chen M.H., Zhang B.Q., You C.H., Lin H.L., Zhao Z.C., Liu M.B., Qiu H.Q., Cheng Y. (2022). Molecular mechanism of *Gelsemium elegans* (Gardner and Champ.) Benth. against neuropathic pain based on network pharmacology and experimental evidence. Front. Pharmacol..

[8] Liu Y.C., Li L., Pi C., Sun Z.L., Wu Y., Liu Z.Y. (2017). Fingerprint analysis of *Gelsemium elegans* by HPLC followed by the targeted identification of chemical constituents using HPLC coupled with quadrupole-time-off light mass spectrometry. Fitoterapia.

[9] Liu Y.C., Xiao S., Yang K., Ling L., Sun Z.L., Liu Z.Y. (2017). Comprehensive identification and structural characterization of target components from *Gelsemium elegans* by high-performance liquid chromatography coupled with quadrupole time-of-flight mass spectrometry based on accurate mass databases combined with MS/MS spectra. J. Mass. Spectrom..

[10] Jin G.L., Su Y.P., Liu M., Xu Y., Yang J., Liao K.J., Yu C.X. (2014). Medicinal plants of the genus *Gelsemium* (Gelsemiaceae, Gentianales)—A review of their phytochemistry, pharmacology, toxicology and traditional use. J. Ethnopharmacol..

[11] Roger A.J., Munoz-Gomez S.A., Kamikawa R. (2017). The origin and diversification of mitochondria. Curr. Biol..

[12] Montilla E.C., Hillebrand S., Winterhalter P. (2011). Anthocyanins in purple sweet potato (*Ipomoea batatas* L.) varieties. Fruit Veg. Cereal Sci. Biotechnol..

[13] Birky C.W. (2001). The inheritance of genes in mitochondria and chloroplasts: Laws, mechanisms, and models. Ann. Rev. Genet..

[14] Wolfe K.H., Li W.H., Sharp P.M. (1987). Rates of nucleotide substitution vary greatly among plant mitochondrial, chloroplast, and nuclear DNAs. Proc. Natl. Acad. Sci. USA.

[15] Wu Z.Q., Liao X.Z., Zhang X.N., Tembrock L.R., Broz A. (2022). Genomic architectural variation of plant mitochondria—A review of multichromosomal structuring. J. Syst. Evol..

[16] Kozik A., Rowan B.A., Lavelle D., Berke L., Schranz M.E., Michelmore R.W., Christensen A.C. (2019). The alternative reality of plant mitochondrial DNA: One ring does not rule them all. PLoS Genet..

[17] Sloan D.B. (2013). One ring to rule them all? Genome sequencing provides new insights into the ‘master circle’ model of plant mitochondrial DNA structure. New Phytol..

[18] Wu Z.Q., Cuthbert J.M., Taylor D.R., Sloan D.B. (2015). The massive mitochondrial genome of the angiosperm Silene noctiflora is evolving by gain or loss of entire chromosomes. Proc. Natl. Acad. Sci. USA.

[19] Zou Y., Zhu W.D., Sloan D.B., Wu Z.Q. (2022). Long-read sequencing characterizes mitochondrial and plastid genome variants in Arabidopsis msh1 mutants. Plant J..

[20] Bi C.W., Paterson A.H., Wang X.L., Xu Y.Q., Wu D.Y., Qu Y.S., Jiang A.N., Ye Q.L., Ye N. (2019). Analysis of the complete mitochondrial genome sequence of the diploid cotton *Gossypium raimondii* by comparative genomics approaches. BioMed Res. Int..

[21] Millar A.H., Heazlewood J.L., Kristensen B.K., Braun H.P., Møller I.M. (2005). The plant mitochondrial proteome. Trends Plant Sci..

[22] Shtolz N., Mishmar D. (2019). The mitochondrial genome–on selective constraints and signatures at the organism, cell, and single mitochondrion levels. Front. Ecol. Evol..

[23] Gras D.E., Mansilla N., Rodríguez C., Welchen E., Gonzalez D.H. (2020). *Arabidopsis thaliana* SURFEIT1-like genes link mitochondrial function to early plant development and hormonal growth responses. Plant J. Cell Mol. Biol..

[24] Jin Y.H., Wu J.J., Peng Y.Q., Hu S.H., Yan H.S., Xiao F., Zhou X., Gao J., Wang R.H., Xu L. (2019). The complete mitochondrial genome of heartbreak grass *Gelsemium elegans* (Gardner & Champ.) Benth. (Gelsemiaceae). Mitochondrial DNA Part B Resour..

[25] Chase C.D. (2007). Cytoplasmic male sterility: A window to the world of plant mitochondrial–nuclear interactions. Trends Genet..

[26] Tang H.W., Zheng X.M., Li C.L., Xie X.R., Chen Y.L., Chen L.T., Zhao X.C., Zheng H.Q., Zhou J.J., Ye S. (2017). Multi-step formation, evolution, and functionalization of new cytoplasmic male sterility genes in the plant mitochondrial genomes. Cell Res..

[27] Toriyama K. (2021). Molecular basis of cytoplasmic male sterility and fertility restoration in rice. Plant Biotechnol..

[28] Melonek J., Small I. (2022). Triticeae genome sequences reveal huge expansions of gene families implicated in fertility restoration. Curr. Opin. Plant Biol..

[29] Sun T., Bentolila S., Hanson M.R. (2016). The unexpected diversity of plant organelle RNA editosomes. Trends Plant Sci..

[30] Gualberto J.M., Mileshina D., Wallet C., Niazi A.K., Weber-Lotfi F., Dietrich A. (2014). The plant mitochondrial genome: Dynamics and maintenance. Biochimie.

[31] Yang Z.J., Ni Y., Lin Z.B., Yang L.B., Chen G.T., Nijiati N., Hu Y.Z., Chen X.Y. (2022). De novo assembly of the complete mitochondrial genome of sweet potato (*Ipomoea batatas* [L.] Lam) revealed the existence of homologous conformations generated by the repeat-mediated recombination. BMC Plant Biol..

[32] Wang S.B., Li D.W., Yao X.H., Song Q.W., Wang Z.P., Zhang Q., Zhong C.H., Liu Y.F., Huang H.W. (2019). Evolution and diversification of kiwifruit mitogenomes through extensive whole-genome rearrangement and mosaic loss of intergenic sequences in a highly variable region. Genome Biol. Evol..

[33] Li J.L., Xu Y.C., Shan Y.Y., Pei X.Y., Yong S.Y., Liu C., Yu J. (2021). Assembly of the complete mitochondrial genome of an endemic plant, *Scutellaria tsinyunensis*, revealed the existence of two conformations generated by a repeat-mediated recombination. Planta.

[34] Alverson A.J., Rice D.W., Dickinson S., Barry K., Palmer J.D. (2011). Origins and recombination of the bacterial-sized multichromosomal mitochondrial genome of cucumber. Plant Cell.

[35] Yu R.X., Sun C.Y., Zhong Y., Liu Y., Sanchez-Puerta M.V., Mower J.P., Zhou R.C. (2022). The minicircular and extremely heteroplasmic mitogenome of the holoparasitic plant Rhopalocnemis phalloides. Curr. Biol..

[36] Guo W., Zhu A., Fan W., Mower J.P. (2017). Complete mitochondrial genomes from the ferns *Ophioglossum californicum* and *Psilotum nudum* are highly repetitive with the largest organellar introns. New Phytol..

[37] Dong S., Zhao C., Chen F., Liu Y., Zhang S., Wu H., Zhang L., Liu Y. (2018). The complete mitochondrial genome of the early flowering plant *Nymphaea colorata* is highly repetitive with low recombination. BMC Genom..

[38] Fang B., Li J.L., Zhao Q., Liang Y.P., Yu J. (2021). Assembly of the complete mitochondrial genome of Chinese plum (*Prunus salicina*): Characterization of genome recombination and RNA editing sites. Genes.

[39] Morgante M., Hanafey M., Powell W. (2002). Microsatellites are preferentially associated with nonrepetitive DNA in plant genomes. Nat. Genet..

[40] Liu Y.C., Liu S., Liu D.C., Wei Y.X., Liu C., Yang Y.M., Tao C.G., Liu W.S. (2014). Exploiting EST databases for the development and characterization of EST-SSR markers in blueberry (*Vaccinium*) and their cross-species transferability in *Vaccinium* spp.. Sci. Hortic..

[41] Cheng Y., He X., Priyadarshani S.V.G.N., Wang Y., Ye L., Shi C., Ye K., Zhou Q., Luo Z., Deng F. (2021). Assembly and comparative analysis of the complete mitochondrial genome of *Suaeda glauca*. BMC Genom..

[42] Christensen A.C. (2013). Plant mitochondrial genome evolution can be explained by DNA repair mechanisms. Genome Biol. Evol..

[43] Smith D.R., Keeling P.J. (2015). Mitochondrial and plastid genome architecture: Reoccurring themes, but significant differences at the extremes. Proc. Natl. Acad. Sci. USA.

[44] Hong Z., Liao X.Z., Ye Y.J., Zhang N.N., Yang Z.J., Zhu W.D., Gao W., Sharbrough J., Tembrock L.R., Xu D.P. (2021). A complete mitochondrial genome for fragrant Chinese rosewood (*Dalbergia odorifera*, Fabaceae) with comparative analyses of genome structure and intergenomic sequence transfers. BMC Genom..

[45] Yang Y.F., Zhu G.N., Li R., Yan S.J., Fu D.Q., Zhu B.Z., Tian H.Q., Luo Y.B., Zhu H.Q. (2017). The RNA editing factor SlORRM4 is required for normal fruit ripening in tomato. Plant Physiol..

[46] He P., Xiao G.H., Liu H., Zhang L.H., Zhao L., Tang M.J., Huang S., An Y.J., Yu J.N. (2018). Two pivotal RNA editing sites in the mitochondrial atp1 mRNA are required for ATP synthase to produce sufficient ATP for cotton fiber cell elongation. New Phytol..

[47] Notsu Y., Masood S., Nishikawa T., Kubo N., Akiduki G., Nakazono M., Hirai A., Kadowaki K. (2002). The complete sequence of the rice (*Oryza sativa* L.) mitochondrial genome: Frequent DNA sequence acquisition and loss during the evolution of flowering plants. Mol. Genet. Genom..

[48] Wang X.C., Chen H., Yang D., Liu C. (2018). Diversity of mitochondrial plastid DNAs (MTPTs) in seed plants. Mitochondrial DNA Part A.

[49] Kitazaki K., Kubo T., Kagami H., Matsumoto T., Fujita A., Matsuhira H., Matsunaga M., Mikami T. (2011). A horizontally transferred tRNA(Cys) gene in the sugar beet mitochondrial genome: Evidence that the gene is present in diverse angiosperms and its transcript is aminoacylated. Plant J. Cell Mol. Biol..

[50] Arseneau J.R., Steeves R., Laflamme M. (2017). Modified low-salt CTAB extraction of high-quality DNA from contaminant-rich tissues. Mol. Ecol. Resour..

[51] Jin J.J., Yu W.B., Yang J.B., Song Y., de Pamphilis C.W., Yi T.S., Li D.Z. (2020). GetOrganelle: A fast and versatile toolkit for accurate de novo assembly of organelle genomes. Genome Biol..

[52] Wick R.R., Schultz M.B., Zobel J., Holt K.E. (2015). Bandage: Interactive visualization of de novo genome assemblies. Bioinformatics.

[53] Li H., Durbin R. (2009). Fast and accurate short read alignment with Burrows-Wheeler transform. Bioinformatics.

[54] Tillich M., Lehwark P., Pellizzer T., Ulbricht-Jones E.S., Fischer A., Bock R., Greiner S. (2017). GeSeq-versatile and accurate annotation of organelle genomes. Nucleic Acids Res..

[55] Lowe T.M., Eddy S.R. (1997). tRNAscan-SE: A program for improved detection of transfer RNA genes in genomic sequence. Nucleic Acids Res..

[56] Chen Y., Ye W., Zhang Y., Xu Y. (2015). High speed BLASTN: An accelerated MegaBLAST search tool. Nucleic Acids Res..

[57] Shi L., Chen H., Jiang M., Wang L., Wu X., Huang L., Liu C. (2019). CPGAVAS2, an integrated plastome sequence annotator and analyzer. Nucleic Acids Res..

[58] Lewis S.E., Searle S., Harris N., Gibson M., Iyer V., Richter J., Wiel C., Bayraktaroglu L., Birney E., Crosby M. (2002). Apollo: A sequence annotation editor. Genome Biol..

[59] Beier S., Thiel T., Münch T., Scholz U., Mascher M. (2017). MISA-web: A web server for microsatellite prediction. Bioinformatics.

[60] Benson G. (1999). Tandem repeats finder: A program to analyze DNA sequences. Nucleic Acids Res..

[61] Stefan K., Choudhuri J.V., Enno O., Chris S., Jens S., Robert G. (2001). REPuter: The manifold applications of repeat analysis on a genomic scale. Nucleic Acids Res..

[62] Zhang H., Meltzer P., Davis S. (2013). RCircos: An R package for Circos 2D track plots. BMC Bioinform..

[63] Zhang D., Gao F., Jakovlić I., Zou H., Zhang J., Li W.X., Wang G.T. (2020). PhyloSuite: An integrated and scalable desktop platform for streamlined molecular sequence data management and evolutionary phylogenetics studies. Mol. Ecol. Resour..

[64] Katoh K., Standley D.M. (2013). MAFFT multiple sequence alignment software version 7: Improvements in performance and usability. Mol. Biol. Evol..

[65] Huelsenbeck J.P., Ronquist F. (2001). MRBAYES: Bayesian inference of phylogenetic trees. Bioinformatics.

[66] Letunic I., Bork P. (2019). Interactive Tree Of Life (iTOL) v4: Recent updates and new developments. Nucleic Acids Res..

[67] Mower J.P. (2009). The PREP suite: Predictive RNA editors for plant mitochondrial genes, chloroplast genes and us-er-defined alignments. Nucleic Acids Res..

