# Supplementary material for: Assembly of the Complete Mitochondrial Genome of Gelsemium elegans Revealed the Existence of Homologous Conformations Generated by a Repeat Mediated Recombination
Source: Int J Mol Sci. 2022 Dec 28;24(1):527. doi: 10.3390/ijms24010527 (PMC9820418; doi:10.3390/ijms24010527)
Supplement: Supplementary file 1 [file ijms-24-00527-s001.zip › ijms-2083533-supplementary.pdf]

SUPPLEMENTARY MATERIALS

Table S1. Primer sequences used in the repeat sequence experiment

Primer name	Primer sequence (5' to 3')
R2 ma1 -F	TGGCTCGCCTTGGTCT
R2 ma1 -R	CACTTAGGAGGATTGGAC
R2 ma2 -F	AAAAGGGTGCTAACGG
R2 ma2 -R	AGGCGCATCAGAAAG
R3 ma1 -F	ATGCCCAAAGACTCCC
R3 ma1 -R	ACCGTCCCGACCAAT
R3 ma2 -F	CCGAAAGAAGGGCGGACAC
R3 ma2 -R	TTTGATAGTAATGGCTGGA
R4 ma1 -F	TAAGAGTGAAATGGTGGGA
R4 ma1 -R	ACGCCGAAACTAAAT
R4 ma2 -F	AGGCGAAACCCAGAA
R4 ma2 -R	CCCTTCGACCCGTTA

Table S2. Path selection for each node based on Oxford Nanopore data

Contig	Type	Path
1	circular	9-84-23-49-16-26-39-75-2-53-7-83-4-61-80-81-82-6-82-1-14-79-13-80-18-77
2	circular	5-15-12-45-38-47-8-28-11-79-78-77-17-83-3-61-80-13-79-14-10-53

Table S3. Microsatellite repeats in the mitogenome of *G. elegans*

Chromosome number	Type	Structure of microsatellite repeats	Size (bp)	Start	End
Chromosome 1	p1	(T)11	11	1787	1797
	p4	(AATG)3	12	3225	3236
	p1	(A)10	10	3808	3817
	p1	(A)10	10	4664	4673
	p4	(TCTT)3	12	7784	7795
	p1	(T)11	11	9878	9888
	p2	(TA)5	10	14,677	14,686
	p1	(A)12	12	15,296	15,307
	p2	(GA)5	10	17,734	17,743
	p1	(A)11	11	27,037	27,047
	p4	(AGAA)3	12	31,822	31,833

p2	(TA)5	10	34,933	34,942
p4	(ATAC)3	12	35,073	35,084
p4	(AGTA)3	12	39,494	39,505
p1	(A)10	10	41,742	41,751
p1	(T)10	10	42,395	42,404
p4	(AAAG)3	12	44,420	44,431
p4	(AAGG)3	12	68,447	68,458
p4	(GGAA)3	12	70,787	70,798
p4	(CTTT)3	12	70,861	70,872
p1	(A)11	11	71,820	71,830
p3	(ATA)5	15	75,101	75,115
p3	(AGA)4	12	79,449	79,460
p1	(A)12	12	84,022	84,033
p1	(A)10	10	86,474	86,483
p1	(T)13	13	94,344	94,356
p2	(AT)6	12	96,693	96,704
p1	(T)11	11	98,021	98,031
p4	(CTTG)3	12	102,218	102,229
p5	(CTTTA)3	15	108,726	108,740
p4	(GGAC)3	12	114,951	114,962
p4	(GCTA)3	12	120,286	120,297
p1	(T)10	10	136,640	136,649
p2	(TA)6	12	139,177	139,188
p4	(GGCA)3	12	142,247	142,258
p3	(CTG)4	12	142,328	142,339
p1	(T)10	10	148,837	148,846
p4	(TCTA)3	12	149,467	149,478
p3	(AGA)4	12	153,212	153,223
p1	(T)11	11	153,428	153,438
p3	(TTA)4	12	158,010	158,021
p4	(GAAA)3	12	158,181	158,192
p4	(CTTT)3	12	158,201	158,212
p1	(G)10	10	158,353	158,362
p2	(CT)5	10	159,914	159,923
p4	(GCAA)3	12	162,366	162,377
p6	(TCCGAG)3	18	163,399	163,416
p1	(T)10	10	164,660	164,669
p1	(A)10	10	164,716	164,725

	p5	(GAAAG)3	15	167,352	167,366
	p1	(T)10	10	171,671	171,680
	p4	(TTTA)3	12	173,461	173,472
	p1	(A)11	11	175,618	175,628
	p2	(CT)5	10	177,340	177,349
	p4	(AAAG)3	12	178,941	178,952
	p4	(GGCG)3	12	182,452	182,463
	p1	(A)10	10	192,284	192,293
	p4	(CAGG)3	12	195,480	195,491
	p4	(AATA)3	12	195,497	195,508
	p2	(TC)5	10	195,789	195,798
	p2	(CT)5	10	198,725	198,734
	p4	(TGAA)3	12	208,416	208,427
	p1	(A)11	11	212,958	212,968
	p3	(TAT)4	12	213,058	213,069
	p2	(CT)5	10	224,828	224,837
	p1	(T)10	10	225,234	225,243
	p5	(CTAGT)3	15	227,889	227,903
	p4	(AATG)3	12	228,516	228,527
	p2	(TC)5	10	233,464	233,473
	p2	(TA)5	10	235,649	235,658
	p4	(TTTC)4	16	235,812	235,827
	p1	(A)11	11	239,539	239,549
	p3	(TTA)4	12	241,591	241,602
	p4	(TCGA)3	12	246,470	246,481
	p2	(AG)5	10	246,589	246,598
	p3	(TCT)4	12	247,900	247,911
	p2	(TA)5	10	248,637	248,646
	p1	(T)11	11	251,327	251,337
	p3	(GAA)4	12	251,609	251,620
	p3	(CAA)4	12	255,885	255,896
	p2	(AT)5	10	256,978	256,987
	p1	(A)10	10	260,745	260,754
Chromosome 2	p1	(A)10	10	1398	1407
	p1	(A)10	10	1528	1537
	p4	(GGCC)3	12	1712	1723
	p4	(TCGC)3	12	2681	2692
	p4	(CTTT)3	12	13,391	13,402

	p4	(AAAG)3	12	14,418	14,429
	p4	(ATAG)3	12	20,219	20,230
	p1	(A)10	10	37,055	37,064
	p3	(TTA)4	12	39,338	39,349
	p3	(CAA)4	12	44,111	44,122
	p2	(AT)5	10	45,204	45,213
	p1	(A)10	10	48,971	48,980
	p2	(AT)5	10	53,351	53,360
	p2	(TA)8	16	53,365	53,380
	p1	(A)12	12	53,408	53,419
	p2	(TC)5	10	53,638	53,647
	p4	(TTAT)3	12	63,229	63,240
	p4	(AAGC)3	12	67,019	67,030
	p4	(TTAA)4	16	72,860	72,875
	p4	(AAGA)3	12	73,822	73,833
	p1	(T)14	14	75,855	75,868
	p4	(TTTC)3	12	78,391	78,402
	p4	(TATC)3	12	84,346	84,357
	p1	(T)12	12	94,046	94,057
	p2	(TC)5	10	95,156	95,165
	p3	(AGA)4	12	95,729	95,740
	p3	(TCT)4	12	95,912	95,923
	p4	(AGAA)3	12	105,777	105,788
	p1	(A)12	12	107,747	107,758
	p1	(T)11	11	110,103	110,113
	p4	(GACC)3	12	111,363	111,374
	p1	(A)14	14	111,810	111,823
	p2	(TA)6	12	115,377	115,388
	p1	(A)14	14	116,328	116,341
	p4	(CATA)3	12	121,,014	121,025
	p1	(T)10	10	124,741	124,750
	p2	(TA)7	14	127,028	127,041
	p3	(AAG)4	12	128,076	128,087
	p2	(TG)5	10	129,108	129,117
	p1	(A)10	10	134,126	134,135
	p3	(GAA)4	12	134,342	134,353
	p4	(AAAG)3	12	142,781	142,792

Table S4 . Tandem repeats in the mitogenome of *G. elegans*

Chromosome number	Start and end sites of the tandem repeats	Period size（bp）	Copy number	Consensus size（bp）	Percent matches	Indels	Score	Bases number				Extropy（0-2）
								A	C	G	T	
Chromosome 1	1,324-1,370	24	2	23	79	4	57	34	23	17	25	1.96
	126,053-126,088	18	2	18	83	0	51	11	16	8	63	1.49
	129,688-129,730	21	2	22	86	4	65	18	13	20	46	1.83
	133,449-133,495	24	2	24	78	0	59	29	34	14	21	1.93
	136,909-136,952	20	2.1	22	83	8	60	52	25	20	2	1.58
	194887-194,947	25	2.4	25	66	0	66	34	18	19	27	1.95
Chromosome 2	40,129-40,161	16	2.1	16	88	5	52	3	12	27	57	1.49
	53,351-53,379	15	1.9	15	92	0	51	44	0	0	55	0.99
	57,293-57,343	26	2	26	84	0	74	68	3	25	1	1.17
	62,115-62,172	27	2.1	27	96	0	109	29	32	15	22	1.95
	97,320-97,364	23	2	23	90	0	76	60	6	8	24	1.51

Table S5. Dispersed repeats in the mitogenome of *G. elegans*

Chromosome number	The repeat length of the first part	The starting site of the first part	Matching direction	The repeat length of the second part	The starting site of the second part	interval distance of repeats	E-value
Chromosome 1	201	123,559	P	201	252,071	-2	3.37×10^{-106}
	132	91,381	P	132	123,521	0	6.50×10^{-70}
	102	5609	F	102	87,799	0	7.50×10^{-52}
	94	91,381	F	94	252,178	0	4.91×10^{-47}
	101	82,571	F	101	155,284	-2	1.36×10^{-46}
	79	22,223	F	79	180,899	-1	1.25×10^{-35}
	78	47,367	P	78	93,737	-3	4.33×10^{-31}
	75	55,241	F	75	246,221	-3	2.46×10^{-29}
	72	143,408	F	72	208,859	-3	1.39×10^{-27}
	65	1235	P	65	228,991	-1	2.76×10^{-27}
	62	111,937	P	62	146,902	-1	1.69×10^{-25}
	62	4728	F	62	215,629	-3	9.25×10^{-22}
	51	1249	P	51	228,991	0	3.80×10^{-21}
	60	143,424	F	60	208,875	-3	1.34×10^{-20}
	56	70,729	F	56	187,020	-2	5.14×10^{-20}
	55	86,837	F	55	216,810	-3	1.05×10^{-17}
	52	89,209	P	52	172,132	-2	1.13×10^{-17}
	52	176,111	P	52	225,884	-2	1.13×10^{-17}
	48	123,712	P	48	252,071	-1	3.50×10^{-17}
	53	189,333	P	53	224,473	-3	1.50×10^{-16}
	50	61,292	P	50	198,359	-2	1.68×10^{-16}
	43	187,404	F	43	212,158	0	2.49×10^{-16}
	40	48,540	F	40	73,503	0	1.59×10^{-14}

40	125,010	F	40	257,265	0	1.59×10^{-14}
39	143,441	F	39	208,892	0	6.38×10^{-14}
45	11,830	P	45	191,134	-2	1.39×10^{-13}
37	4364	F	37	181,810	0	1.02×10^{-12}
40	70,745	F	40	187,036	-1	1.91×10^{-12}
43	4747	F	43	215,648	-2	2.02×10^{-12}
36	55,280	F	36	246,260	0	4.08×10^{-12}
45	17,748	P	45	42,626	-3	5.96×10^{-12}
39	172,825	F	39	228,217	-1	7.46×10^{-12}
40	172,892	F	40	228,391	-2	1.12×10^{-10}
33	87,136	P	33	229,103	0	2.61×10^{-10}
42	41,010	F	42	215,942	-3	3.09×10^{-10}
36	82,487	F	36	241,765	-1	4.41×10^{-10}
41	16,810	F	41	248,438	-3	1.15×10^{-09}
41	40,988	F	41	215,920	-3	1.15×10^{-09}
41	124,965	F	41	253,515	-3	1.15×10^{-09}
31	70,754	F	31	187,045	0	4.18×10^{-09}
31	123,842	P	31	125,861	0	4.18×10^{-09}
31	205,518	P	31	216,590	0	4.18×10^{-09}
40	125,708	F	40	258,328	-3	4.25×10^{-09}
34	6106	P	34	94,591	-1	6.66×10^{-09}
30	88,860	F	30	228,028	0	1.67×10^{-08}
30	98,044	F	30	257,672	0	1.67×10^{-08}
36	75,534	F	36	91,340	-2	2.31×10^{-08}
33	4757	F	33	215,658	-1	2.59×10^{-08}
33	5625	F	33	111,851	-1	2.59×10^{-08}
33	87,815	F	33	111,851	-1	2.59×10^{-08}
38	41,002	F	38	215,934	-3	5.81×10^{-08}
35	86,857	F	35	216,830	-2	8.74×10^{-08}
32	74,903	P	32	110,810	-1	1.00×10^{-07}
32	124,884	F	32	257,148	-1	1.00×10^{-07}
32	175,540	P	32	246,534	-1	1.00×10^{-07}
34	47,411	P	34	93,737	-2	3.30×10^{-07}
34	189,352	P	34	224,473	-2	3.30×10^{-07}
31	5790	P	31	257,142	-1	3.89×10^{-07}
31	74,935	P	31	211,274	-1	3.89×10^{-07}
31	122,997	F	31	208,866	-1	3.89×10^{-07}
36	147,788	P	36	170,927	-3	7.87×10^{-07}
30	12,583	P	30	79,550	-1	1.50×10^{-06}
30	22,003	P	30	242,408	-1	1.50×10^{-06}
32	182,456	P	32	238,522	-2	4.66×10^{-06}
34	24,519	P	34	110,969	-3	1.05×10^{-05}
34	31,155	P	34	242,412	-3	1.05×10^{-05}
31	20,618	P	31	114,661	-2	1.75×10^{-05}
31	89,233	P	31	172,129	-2	1.75×10^{-05}
31	90,198	F	31	104,863	-2	1.75×10^{-05}
31	122,997	F	31	143,415	-2	1.75×10^{-05}
31	179,496	P	31	256,816	-2	1.75×10^{-05}
33	20,402	P	33	248,503	-3	3.85×10^{-05}
33	36,053	P	33	98,036	-3	3.85×10^{-05}
33	41,030	F	33	215,962	-3	3.85×10^{-05}
33	98,922	F	33	249,591	-3	3.85×10^{-05}
33	124,923	F	33	257,207	-3	3.85×10^{-05}
30	7223	F	30	181,187	-2	6.54×10^{-05}
30	24,583	F	30	251,068	-2	6.54×10^{-05}
30	35,613	P	30	179,985	-2	6.54×10^{-05}

	30	125,721	F	30	258,341	-2	6.54×10^{-05}
	30	136,587	F	30	238,102	-2	6.54×10^{-05}
	30	175,458	P	30	228,031	-2	6.54×10^{-05}
	32	13,354	F	32	219,519	-3	1.40×10^{-04}
	32	24,679	F	32	251,082	-3	1.40×10^{-04}
	32	90,090	F	32	104,760	-3	1.40×10^{-04}
	32	123,107	F	32	180,575	-3	1.40×10^{-04}
	32	124,927	F	32	253,477	-3	1.40×10^{-04}
	32	239,163	F	32	251,145	-3	1.40×10^{-04}
	31	5725	F	31	91,235	-3	5.07×10^{-04}
	31	25,930	F	31	187,408	-3	5.07×10^{-04}
	31	25,930	F	31	212,162	-3	5.07×10^{-04}
	31	90,791	P	31	171,983	-3	5.07×10^{-04}
	31	147,753	P	31	170,963	-3	5.07×10^{-04}
	31	172,839	F	31	228,231	-3	5.07×10^{-04}
	30	28,298	F	30	119,695	-3	1.83×10^{-03}
	30	31,568	F	30	57,449	-3	1.83×10^{-03}
	30	34,115	P	30	131,904	-3	1.83×10^{-03}
	30	60,065	P	30	238,221	-3	1.83×10^{-03}
	30	94,794	P	30	256,816	-3	1.83×10^{-03}
	30	179,497	F	30	217,150	-3	1.83×10^{-03}
	30	187,409	P	30	249,598	-3	1.83×10^{-03}
	30	189,634	F	30	221,537	-3	1.83×10^{-03}
	30	212,163	P	30	249,598	-3	1.83×10^{-03}
	30	224,355	P	30	253,578	-3	1.83×10^{-03}
Chromosome 2	163	166	F	163	45,368	0	4.28×10^{-89}
	73	14,232	F	73	61,679	0	6.56×10^{-35}
	74	53,180	P	74	134,639	-3	2.87×10^{-29}
	64	32,746	F	64	89,640	-1	3.30×10^{-27}
	70	53,166	P	70	134,657	-3	6.21×10^{-27}
	52	45,284	F	52	77,351	-3	1.72×10^{-16}
	44	14,271	F	44	109,879	-1	2.50×10^{-15}
	40	78,574	F	40	125,848	0	4.84×10^{-15}
	48	53,212	P	48	134,633	-3	3.45×10^{-14}
	48	53,224	P	48	134,621	-3	3.45×10^{-14}
	46	28,758	F	46	102,644	-3	4.84×10^{-13}
	36	28,768	F	36	102,654	0	1.24×10^{-12}
	36	54,544	F	36	61,829	0	1.24×10^{-12}
	36	78,547	P	36	125,821	0	1.24×10^{-12}
	34	61,718	F	34	109,879	0	1.98×10^{-11}
	32	31,724	F	32	140,300	0	3.17×10^{-10}
	38	110,742	F	38	110,798	-2	4.90×10^{-10}
	39	53,237	P	39	134,617	-3	4.78×10^{-09}
	32	23,881	P	32	46,148	-1	3.05×10^{-08}
	37	98,171	F	37	101,223	-3	6.50×10^{-08}
	31	62,114	F	31	62,141	-1	1.18×10^{-07}
	30	17,929	P	30	45,041	-1	4.57×10^{-07}
	30	45,040	F	30	109,893	-1	4.57×10^{-07}
	32	45,309	F	32	77,376	-2	1.42×10^{-06}
	34	41,721	F	34	77,472	-3	3.20×10^{-06}
	31	14,285	F	31	45,040	-2	5.31×10^{-06}
	33	14,235	F	33	33,640	-3	1.17×10^{-05}
	33	33,640	F	33	61,682	-3	1.17×10^{-05}
	30	3828	P	30	3828	-2	1.99×10^{-05}
	30	13,744	F	30	109,858	-2	1.99×10^{-05}
	30	17,931	P	30	109,892	-2	1.99×10^{-05}

	30	37,013	P	30	77,442	-2	1.99×10^{-05}
	32	4175	F	32	45,891	-3	4.25×10^{-05}
	32	15,148	F	32	110,427	-3	4.25×10^{-05}
	31	14,237	F	31	109,845	-3	1.54×10^{-04}
	31	61,684	F	31	109,845	-3	1.54×10^{-04}
	30	187	F	30	77,421	-3	5.56×10^{-04}
	30	14,286	P	30	17,929	-3	5.56×10^{-04}
	30	15,977	F	30	111,748	-3	5.56×10^{-04}
	30	45,389	F	30	77,421	-3	5.56×10^{-04}
	30	72,887	P	30	121,304	-3	5.56×10^{-04}

Table S6. The homologous DNA fragment in the mitogenome of *G. elegans*

Number	Mitochondrial genome	Identity (%)	Alignment length	Mismatches	Gap openings	Alignment start (chloroplast genome)	Alignment end (chloroplast genome)	MTPT annotation
1	Chromosome 1	98.986	1578	10	1	129,670	131,247	Complete (*trn*N-GUU)
2	Chromosome 1	98.986	1578	10	1	109,228	110,805	Complete (*trn*N-GUU)
3	Chromosome 1	96.552	1073	32	4	94,082	95,151	Partial (*ycf*2)
4	Chromosome 1	96.552	1073	32	4	145,324	146,393	Partial (*ycf*2)
5	Chromosome 1	94.615	520	17	4	29,354	29,868	Complete (*pet*N)
6	Chromosome 1	100	382	0	0	133,453	133,834	Partial (*rrn*23S)
7	Chromosome 1	100	382	0	0	106,641	107,022	Partial (*rrn*23S)
8	Chromosome 1	87.189	562	52	9	46,718	47,272	Partial (*rps*4)
9	Chromosome 1	94.149	376	16	5	34,363	34,732	Partial (*psb*D)
10	Chromosome 1	82.068	619	56	27	44,689	45,299	Partial (*ycf*3)
11	Chromosome 1	80.46	522	85	13	72,057	72,571	Partial (*clp*P)
12	Chromosome 1	73.874	888	179	38	137,821	138,684	Partial (*rrn*16S)
13	Chromosome 1	73.874	888	179	38	101,791	102,654	Partial (*rrn*16S)
14	Chromosome 1	97.661	171	3	1	31,673	31,843	Complete (*trn*D-GUC)
15	Chromosome 1	89.32	206	13	4	46,303	46,508	Complete (*trn*S-GGA)
16	Chromosome 1	87.73	163	11	6	31,649	31,802	Complete (*trn*D-GUC)
17	Chromosome 1	85.38	171	16	3	5319	5489	Partial (*rps*16)
18	Chromosome 1	98.507	67	1	0	150,053	150,119	Partial (*ycf*2)
19	Chromosome 1	98.507	67	1	0	90,356	90,422	Partial (*ycf*2)
20	Chromosome 1	94.737	76	4	0	130,614	130,689	Partial (*trn*N-GUU)
21	Chromosome 1	94.737	76	4	0	109,786	109,861	Partial (*trn*N-GUU)
22	Chromosome 1	98.148	54	1	0	135,002	135,055	IGS (*rrn*23S, *trn*A-UGC)
23	Chromosome 1	98.148	54	1	0	105,420	105,473	IGS (*trn*A-UGC, *rrn*23S)
24	Chromosome 1	78.409	88	18	1	8965	9052	Complete (*trn*S-GCU)
25	Chromosome 2	99.801	4529	9	0	96,682	101,210	Complete (*rps*7), Partial (*ndh*B)
26	Chromosome 2	99.801	4529	9	0	139,265	143,793	Complete (*rps*7), Partial

								(*ndh*B)
27	Chromosome 2	99.268	1229	8	1	35,579	36,807	Partial (*psb*C)
28	Chromosome 2	99.477	1147	6	0	17,310	18,456	Partial (*rpo*C2)
29	Chromosome 2	85.333	225	27	5	67,749	67,970	Complete (*trn*W-CCA)
30	Chromosome 2	91.603	131	10	1	36,203	36,332	Partial (*psb*C)
31	Chromosome 2	97.333	75	2	0	1	75	Complete (*trn*H-GUG)
32	Chromosome 2	100	53	0	0	103,197	103,249	IGS (*rrn*16S, *trn*I-GAU)
33	Chromosome 2	100	53	0	0	137,226	137,278	IGS (*trn*I-GAU, *rrn*16S)
34	Chromosome 2	100	41	0	0	121,216	121,256	Partial (*ndh*A)
35	Chromosome 2	94.872	39	2	0	35,501	35,539	Partial (*psb*C)
36	Chromosome 2	100	31	0	0	143,721	143,751	Partial (*ndh*B)
37	Chromosome 2	100	31	0	0	96,724	96,754	Partial (*ndh*B)
